# The role of mitochondrial dynamics in disease

**DOI:** 10.1002/mco2.462

**Published:** 2023-12-28

**Authors:** Yujuan Wang, Xinyan Dai, Hui Li, Huiling Jiang, Junfu Zhou, Shiying Zhang, Jiacheng Guo, Lidu Shen, Huantao Yang, Jie Lin, Hengxiu Yan

**Affiliations:** ^1^ Immunotherapy Laboratory Qinghai Tibet Plateau Research Institute Southwest Minzu University Chengdu Sichuan China; ^2^ Immunotherapy Laboratory College of Pharmacology Southwest Minzu University Chengdu Sichuan China

**Keywords:** context, disease, mitochondrial dynamics, mitophagy, target treatment

## Abstract

Mitochondria are multifaceted and dynamic organelles regulating various important cellular processes from signal transduction to determining cell fate. As dynamic properties of mitochondria, fusion and fission accompanied with mitophagy, undergo constant changes in number and morphology to sustain mitochondrial homeostasis in response to cell context changes. Thus, the dysregulation of mitochondrial dynamics and mitophagy is unsurprisingly related with various diseases, but the unclear underlying mechanism hinders their clinical application. In this review, we summarize the recent developments in the molecular mechanism of mitochondrial dynamics and mitophagy, particularly the different roles of key components in mitochondrial dynamics in different context. We also summarize the roles of mitochondrial dynamics and target treatment in diseases related to the cardiovascular system, nervous system, respiratory system, and tumor cell metabolism demanding high‐energy. In these diseases, it is common that excessive mitochondrial fission is dominant and accompanied by impaired fusion and mitophagy. But there have been many conflicting findings about them recently, which are specifically highlighted in this view. We look forward that these findings will help broaden our understanding of the roles of the mitochondrial dynamics in diseases and will be beneficial to the discovery of novel selective therapeutic targets.

## Graphical abstract

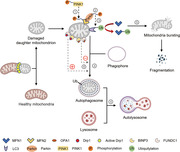

## 1 INTRODUCTION

Mitochondria are primarily known as the “powerhouses” of cells, providing 80% of the energy required for normal cellular function through oxidative phosphorylation (OXPHOS).^1^, ^2^ They are multifaceted organelles regulating various important cellular processes, such as cell cycle, signal transduction, autophagy, metabolism, and determining cell fate during differentiation, maintenance of calcium homeostasis, innate immunity including anti‐coronaviruses immunity.^3^, ^4^, ^5^, ^6^, ^7^, ^8^ Moreover, mitochondria become highly dynamic by undergoing a fusion–fission cycle referred to as mitochondrial dynamics and constant changes in number and morphology in response to various environmental cues.^9^, ^10^ As important parts of mitochondrial quality control, mitochondrial division and fusion are two opposite but coordinated processes, that determine the shapes and number of mitochondria and affect their function.^2^, ^5^, ^11^, ^12^ Another mitochondrial quality control is the selective removal of damaged or aging mitochondria. This process coordinates mitochondrial dynamics to maintain cellular homeostasis, although these processes independent processes.^5^, ^13^ Mitochondria are heterogeneous, and their morphology and function govern adaptation to an ever‐changing cellular environment and are subject to sophisticated regulation.^2^, ^14^ Thus, identifying cellular processes important for cell growth or cytopathy affected by mitochondrial dynamics changes remains a challenge and an important topic in mitochondrion‐related research.

Given the important role of mitochondria in cellular homeostasis, disturbing mitochondrial dynamics is unsurprisingly related with the development of various diseases, especially diseases occurring in energy‐sensitive systems, including cardiovascular diseases (CVDs), neurodegenerative disorders, cancer, and lung disorders.^2^, ^7^, ^9^, ^15^ These diseases have serious impacts on people's lives and health, and scientists are committed to researching mechanisms to find ideal therapeutic interventions. In recent decades, research into the roles of mitochondrial dynamics in human diseases have been extensively studied, which are extremely complex, even contradictory, heavily depending on surrounding environments and stimuli.^7^, ^16^, ^17^, ^18^, ^19^ Therefore, the dysfunction of mitochondrial dynamic promotes or inhibits the occurrence or development of these pathologies.

In this review, we summarize recent developments in our understanding of the molecular biology of mitochondrial dynamics and mitophagy and discuss how imbalances in mitochondrial dynamics lead to abnormal cellular processes and their role in a broad variety of disorders, such as CVDs, neurodegenerative disorders, cancer, and lung disorders. We then summarize drugs and interventions that target mitochondrial dynamics for the treatment of the diseases, emphasizing the importance of mitochondrial dynamics as an emerging therapeutic target. We specifically highlight conflicting results in these studies and hope that these findings will help broaden our understanding of the roles of the mitochondrial dynamics in diseases and will be beneficial to the discovery of novel selective therapeutic targets.

## 2 MITOCHONDRIAL DYNAMICS IN HEALTH

**FIGURE 1:**
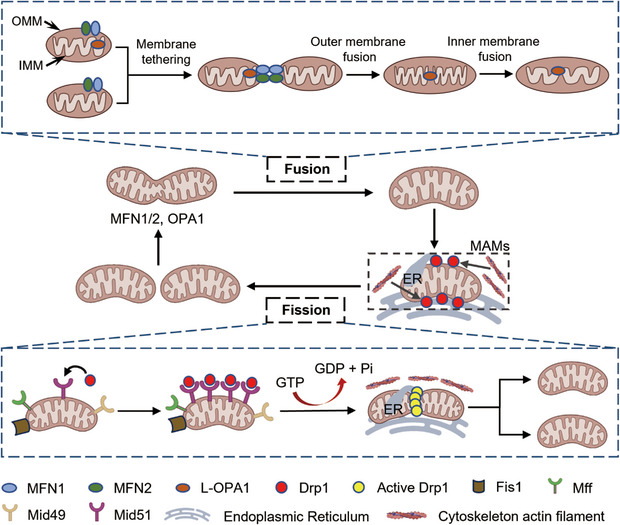
Mitochondrial dynamics: fusion and fission. Mitochondrial fusion and fission are two opposite but coordinated processes. Mitochondrial fusion is orchestrated by three main dynamin‐related GTPases proteins: mitofusin1 (MFN1), mitofusin2 (MFN2), and optic atrophy 1 (OPA1). Upon GTP binding and hydrolysis, conformational changes in the GTPase domains of MFN1 lead to their oligomerization, facilitating the clustering of the two OMMs and subsequent fusion. OPA1 is needed in only one of two colliding mitochondria for fusing the inner membranes of adjacent mitochondria. Mitochondrial fission begins with the labeling of mitochondrial membranes by ER and ER‐mitochondrial contact sites (MAMs), which induces cytoplasmic DRP1 to be recruited to the mitochondrial outer membrane, where it binds to its adapters FIS1, MFF, MiD49, and MiD51 to form a ring structure to complete the entire fission process. In addition, the cytoskeleton actin filaments at MAMs promote DRP1 recruitment and stimulate its GTPase activity.

Mitochondrial dynamics is an indispensable part of adapting to various changes in the cell environment, resulting in cell homeostasis and preventing disease, which refers to the continuous remodeling of the cellular mitochondrial network in a series of processes, including cell content fission and fusion, and ultrastructural reshaping of the membrane.^5^, ^8^ Fusion and fission are key and opposing events controlling mitochondrial dynamics that need to be balanced to support normal mitochondrial and cell function (Figure 1).^8^ Balance between fusion and fission is achieved through the regulation of several dynamics‐related proteins located in the inner mitochondrial membrane (IMM) and outer mitochondrial membrane (OMM), which contain highly conserved GTPase domains and exhibit the ability to self‐assemble, hydrolyze guanosine triphosphate (GTP), and reshape membranes.^20^

### 2.1 Mitochondrial fission

#### 2.1.1 Mechanisms of mitochondrial fission

Dynamin‐related protein 1 (DRP1) is the key mediator of mitochondrial fission, belongs to GTP‐hydrolyzing enzyme. Physiologically, DRP1 is located in the cytoplasm. Under stress, cytosolic DRP1 monomers are recruited to the OMM by the four main adaptor proteins, involving mitochondrial fission factor (MFF), mitochondrial fission 1 protein (FIS1), and mitochondrial dynamics proteins (MIDs) 49 and 51 used to anchor DRP1 to the OMM. Mechanistic studies about DRP1‐mediated scission through cryo‐electron microscopy analysis of MID49/MID51–DRP1–GTP complexes disclosed that GTP binding to DRP1 facilitates the formation of DRP1 linear oligomers. Subsequent GTP hydrolysis shortens while curling associating with polymer into helical ring closed rings with an inner diameter of 16 nm leading to the breakdown of the OMM and IMM, and finally triggering mitochondrial fission.^8^, ^21^ Indeed, a growing body of evidence suggests that abnormal mitochondrial fission directly contributes to disease development.^5^, ^7^, ^22^, ^23^ Mitochondrial fission is not an autonomous process, is also associated with the actin cytoskeleton, endoplasmic reticulum (ER), Golgi apparatus and lysosomes. Fission processing takes place at ER and ER‐mitochondrial contact sites or mitochondria–ER association membranes (MAMs), in which ER might function as a platform for DRP1 oligomerization^7^, ^24^, ^25^ and ER tubules wrap around the mitochondria to promote OMM constriction.^7^, ^26^ The sites of mitochondrial fission seem to be related with the dynamics of mitochondrial DNA (mtDNA), and most fission events occur close to nucleoids compressed from mtDNA in wild‐type cells.^27^ In addition, DRP1 recruitment and its GTPase activity have been promoted by the cytoskeleton actin filaments at MAMs.^7^, ^28^, ^29^ As reported in a study published in 2022, DRP1 is retrograde transported from peripheral cytoplasm to distal mitochondria in the perinuclear region along actin filaments.^30^

As a central mediator, DRP1 regulates itself anchoring, polymerization, and activation for adapting to different cellular context by multiple posttranslational modifications for example phosphorylation‐dephosphorylation, S‐nitrosylation, SUMOylation,^31^ O‐GlcNAcylation,^32^ and ubiquitination.^33^ DRP1 has two key phosphorylation sites with opposite effects. One stimulates (Ser616, Ser579, and Ser600), whereas the other inhibits (Ser637 and Ser656) mitochondrial fission upon phosphorylation by PKA, AMPK, SIRT, and other singals.^8^, ^21^, ^34^ However, in podocytes DRP1 phosphorylation at Ser637/656 promotes mitochondrial fission due to high glucose conditions,^9^, ^35^ and Ser600 phosphorylation by PKA decreases DRP1 GTPase activity.^36^ In a study published in 2021, DRP1 phosphorylation at Ser637 was shown to promote subsequent Ser616 phosphorylation, and only blocking downstream Ser616 phosphorylation leads to mitochondrial elongation rather than fission.^37^ In mouse embryonic fibroblasts, phosphorylation at both sites is essential for maximal fission, indicating that phosphorylation at Ser637 promotes fission or fusion and this function depends on Ser616 phosphorylation status.^37^ SUMO1, SUMO2, or SUMO3 have opposite effects on DRP1 activity. SUMO2 and SUMO3 reduce DRP1 association with OMM,^38^ whereas the conjugation of SUMO1 promotes DRP1 binding,^39^ DRP1 posttranslational modifications are clearly complex and exist depending on the cellular context. No single posttranslational modification of DRP1 is likely to determine the physiological or pathological effects. Conversely, multiple posttranslational modifications probably exert collaborative or additive effect. Therefore, the simultaneous evaluation of multiple posttranslational modifications in a given cellular environment can contribute to the proper understanding of the function of DRP1 in a specific cellular activity.

Each adaptor protein can independently recruit DRP1 to mitochondria to initiate fission, and MFF and MIDs physically interact with each other in a DRP1 complex to coordinate fission.^40^, ^41^ MFF, MID49, and MID51 are directly associated with mitochondrial division, and their absence leads to significant mitochondrial elongation.^40^, ^42^ The role of MFF is relatively simple, its overexpression enhances mitochondrial fragmentation, and its low expression inhibits DRP1 anchor to mitochondria and results in elongated mitochondria. Compared with MFF, MID has a more complex mode of action. A low MID level enhances mitochondrial fission, but MID overexpression leads to the elongation of mitochondria.^43^ Some studies have suggested that MID overexpression recruits inactive DRP1, which in turn stimulates rapid mitochondrial cleavage when subjected to other stimuli.^21^, ^44^ And MID51 can inhibit the enhancement of GTPase activity of DRP1 stimulated by MFF.^40^ As the first reported mitochondrial adaptor protein, FIS1 plays a key role in yeast mitochondrial fission, but its role is less significant in mammalian cells.^7^, ^45^, ^46^ Mammalian cells lacking FIS1 show little or no fission defect.^7^, ^47^ Instead, FIS1 is necessary for some forms of mitophagy.^7^, ^48^, ^49^, ^50^ In 2021, FIS1 was reported to recruit DRP1 to participate in mitochondrial asymmetrical fission, whereas MFF seems to be uniquely related to symmetrical fission.^49^

Dynamin protein 2 and BAX interacting factor 1 help DRP1 to mediate mitochondrial fission.^51^ Ganglioside‐induced differentiation‐associated protein 1,^52^ death‐associated protein 3,^53^ and the aptly‐named mitochondrial fission process 1 (MTFP1/MTP18)^54^, ^55^, ^56^ have also been reported to be involved in mitochondrial fission process. Cardiolipin (CL) interacts with DRP1 to drive the oligomerization of DRP1 and stimulate its GTPase activity to enhance the constriction of mitochondrial OMM and IMM.^57^

#### 2.1.2 Role in mitochondrial quality control

In response to mitochondrial insults, cells have developed a mitochondrial quality control machinery to maintain the mitochondrial integrity and function, including mitochondrial fission/fusion, mitophagy, and mitochondrial biogenesis.^5^, ^58^, ^59^

In physiological conditions, mitochondria undergo continuous cycles of fusion and fission to maintain mitochondrial morphology and number. Mitochondrial fission is an important physiological process accompanying mitosis, characterized by the division of one mitochondrion into two daughter mitochondria, including symmetrical and asymmetrical division. Symmetrical mitochondrial fission is closely orchestrated with the cell cycle to promote the equal segregation of two functional daughter mitochondria during cell division, known as replicative fission.^10^, ^60^ During the cell cycle, mitochondria and cytoskeletal components work together to promote the cell dichotomy and equal mitochondrial separation into two daughter cells, including cytosolic contents and mtDNA. Studies have shown that the proliferation inhibition of various cells is affected by the loss of DRP1, such as vascular smooth muscle cells, myofibroblasts, and pulmonary artery smooth muscle cells,^61^, ^62^ demonstrating the importance of mitochondrial fission in cell division. Moreover, defects in fission lead to the formation of abnormally enlarged mitochondrial DNA nucleoids that cluster in the fused mitochondria, which correlates with the occurrence and development of many diseases caused by the disruption of mitochondrial respiration.^27^

Another form of fission is asymmetrical division that results in one healthy mitochondrion and one senile, dysfunctional, small fragment. The small, dysfunctional fragment is first prevented from fusing with other mitochondria, then subsequently engulfed and degraded by mitochondrial autophagy (mitophagy).^9^, ^33^ During mitophagy, a double‐membraned autophagosome is initially formed, and then the dysfunctional mitochondria are engulfed and delivered to the lysosome for degradation by hydrolytic enzymes.^63^ DRP1‐mediated fission can promote mitophagy. The inhibition of DRP1 reduces Parkin‐mediated mitophagy,^64^ and mutations in DRP1 results in fatal heart failure because of defects in mitophagy.^65^ DRP1 interacts with the zinc transporter ZIP1 to promote the flow of positively charged Zn2+ into the mitochondria matrix, causing mitochondrial membrane depolarization, which results in OMA1 activation and OPA1 inactivation and ultimately enables the clearance of damaged mitochondrial fragments through mitophagy.^66^, ^67^ However, the daughter mitochondria derived from symmetrical fission maintain membrane polarization and re‐​fuse with the mitochondrial network.^49^, ^67^ Notably, evidence has shown that DRP1 plays an important role in promoting mitochondrial autophagy, but some reports have indicated that DRP1 is not necessary for mitophagy. For example, in cardiac‐​specific DRP1‐​knockout mice, preventing mitophagy delays the progression of cardiomyopathy.^68^

### 2.2 Mitochondrial fusion

Mitochondrial fusion is the connection of two organelles in a tubular network, which is closely related to the exchange of contents, reduction in the concentration of superoxide and mutated mtDNA, and maintenance of genetic and biochemical homogeneity.^69^, ^70^ It is a multistep process with a specific sequence: ① membrane tethering, ② OMM fusion, and ③ IMM fusion.^2^ In a typical mitochondrial fusion reaction, after two mitochondria collide, the membrane fusion event occurs at the site of collision, beginning with OMM fusion followed by IMM fusion and ending in matrix exchange.^2^, ^7^, ^22^, ^23^ A kiss‐and‐run form of fusion has been observed in cultured rat cardiac myocytes, in which only exchange of matrix proteins occurs without obvious merging or structural rearrangement when two mitochondria transiently interact. Mitochondrial fusion is mainly involved in the formation of new mitochondria and the renovation of dysfunctional mitochondria, such as mtDNA mutation and membrane potential reduction.^8^, ^12^, ^57^ For example, mitochondrial with dysfunction fuse with normal mitochondria, mtDNA is reintegrated and renewed, and mitochondrial membrane potential (MMP) returns to normal levels.^71^ When an organism is in a state of stress (e.g., disease, hunger, and hypoxia), mitochondrial fusion maximizes the generation of ATP, supporting the energy needs of an organism.^72^ This process facilitates the elongation of the mitochondrial network by enhancing fusion or inhibiting mitochondrial fission. Meanwhile, maintaining a highly fused network of mitochondria by limiting fission rate is conducive to organelles not being degraded by autophagy during starvation.

#### 2.2.1 Mechanisms of mitochondrial fusion

Mitochondrial fusion is orchestrated by three main dynamin‐related GTPases proteins: mitofusin1 (MFN1), mitofusin2 (MFN2), and optic atrophy 1 (OPA1).^8^, ^22^, ^57^ MFN1 and MFN2 are OMM transmembrane GTPases required for outer membrane fusion, whereas OPA1 mostly mediates mitochondrial inner membrane fusion.^73^ MFN1 and MFN2 are highly homologous and have similar structural organization,^8^ which exert redundant functions. Low expression of MFN1 may be compensated by MFN2.^22^ MFN1 and OPA1 are core components of fusion, but the exact role of MFN2 in fusion is unclear. MFN1 is a typical GTP enzyme. When MFN1 binds to GTP, GTP is hydrolyzed and conformational changes in the MFN1's GTPase domains lead to its oligomerization, promoting the clustering of the two OMMs and subsequent fusion.^8^ A new mechanism for the oligomerization of mitofusin (MFN) molecules was proposed by Mattie et al, who demonstrated that their oligomerization arise through the heptad repeat 2 (HR2) domains in MFN oxidized by oxidized glutathione, suggesting that redox signaling plays a vital role in OMM fusion.^57^, ^74^ MFN2, encoded by the *MFN2* gene, plays a role in the regulation of mitochondrial fusion. However, MFN2 is now thought to play other roles in mitophagy, mitochondrial movement, and lipid transfer and is a link to other organelles, including the ER, lysosome, peroxydasis, endosome, and lipid droplets.^8^ The tether action of MFN2 is a vital mediator of mitochondrion–ER contact sites, which regulate the many important mitochondrial functions, such as calcium homeostasis and lipid metabolism.^2^, ^75^, ^76^ The knockout of either MFN1 or MFN2 in mice leads to embryonic lethality,^7^ and the overexpression of MFN1 or MFN2 in embryo fibroblasts cells from MFN2‐KO or MFN1‐KO mouse restore mitochondrial fusion.^2^ Intriguingly, cardiac‐specific MFN1‐ or MFN2‐knockout mice develop normally, although cardiac‐specific deletion of MFN1–MFN2 causes rapid heart failure and premature death in mice. MFN1 and MFN2 are essential to fusion but play different roles.^7^

Unlike mitofusins, OPA1 is needed in only one of two colliding mitochondria for fusing the inner membranes of adjacent mitochondria^8^ and consists of eight isoforms forming one to three proteolytic cleavage sites, namely, S1, S2, and 15‐oxospiramilactone (S3), depending on the set of exons 4, 4b, and 5b.^22^, ^57^ The proteolysis of OPA1 is mediated by various proteins: mitochondrial metalloendopeptidase OMA1, ATP‐dependent zinc metalloprotease YME1L1, presenilin‐associated rhomboid‐like (PARL) protein, paraplegin, and mAAA protease complex ATPase family gene‐3 yeast‐like‐1.^22^ Apart from proteolytic cleavage, increased level of OXPHOS promotes the cleavage of OPA1 by YME1L.^25^ Different forms of OPA1 function remains open.^8^ Only isoforms that produce long and short isoforms can restore mitochondrial fusion defects in mouse OPA1‐null cells. If the S1 site is not hydrolyzed but remains intact, the resulting long‐form of OPA1 (L‐OPA1) is recruited to the IMM, which has been shown to promote mitochondrial fusion by itself.^57^ Under certain conditions associated with stress, the S1 site is cleaved by YME1L1 or OMA1 to produce a short form of OPA1 (S‐OPA1),^8^, ^25^, ^57^ which do not have a membrane anchor but can still modulate fusion activity by forming complexes with the membrane‐bound L‐OPA1 isoforms. For example, the membrane fusion activity of L‐OPA1 in vitro is enhanced by the addition of S‐OPA1.^77^ In general, a coordination of L‐OPA1 and S‐OPA1 is required for the normal levels of mitochondrial fusion under basal conditions. Notably, S‐OPA1 alone mediates GTP‐dependent fusion when added to liposomes, such as CL, which is a phospholipid that makes up the IMM and vital for IMM fusion by taking part in the assembly and stability of large protein complexes, including mitochondrial contact site and cristae organizing system, as well as OXPHOS complexes.^57^, ^77^, ^78^ However, in contrast to with these results, S‐OPA1 helps to shifting mitochondrial dynamics toward fission and fragmentation on mitochondrial dysfunction.^8^, ^22^ Processing OPA1 has extensive impact on mitochondrial dynamics, and the appropriate balance between long and short L‐OPA1 isoforms is critical for fusion, but how different forms of OPA1 function requires in‐depth study. In addition to regulating IMM fusion, OPA1 participates in cristae remodeling.

#### 2.2.2 Mitochondrial network formation and homeostasis

In physiological state, mitochondrial homeostasis is regulated by mitochondrial network formation including mitochondrial biogenesis, fission, fusion, and mitophagy that preserve organelle structure and function.^13^, ^60^, ^79^ Mitochondrial biogenesis is defined as the process of increasing the number of mitochondria from the growth and fission of preexisting mitochondria.^80^, ^81^ Cumulative evidence indicates that mitophagy plays a key role in mitochondrial homeostasis^48^, ^82^ especially in the coronavirus disease 2019 (COVID‐19).^83^

**FIGURE 2:**
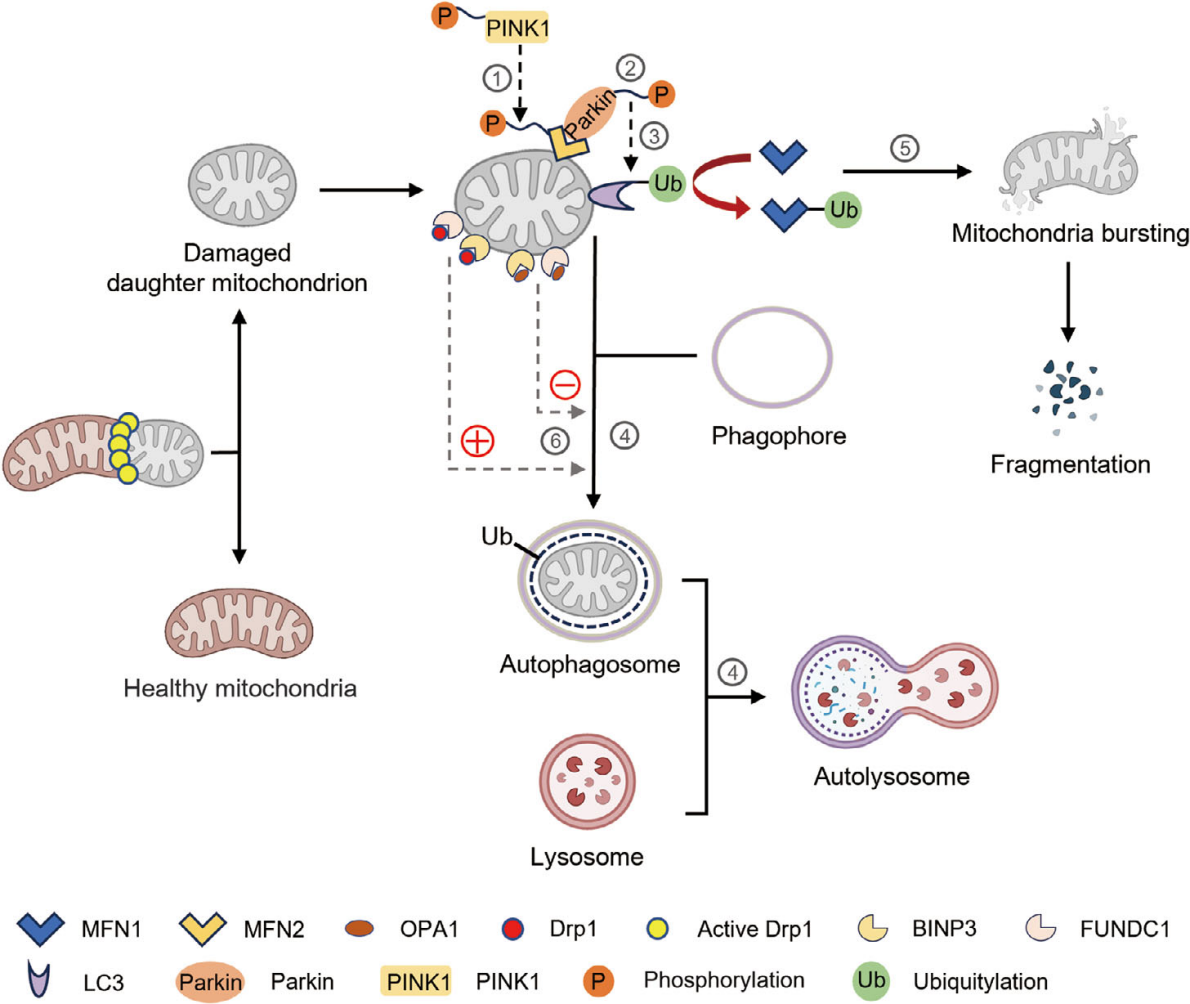
Interaction between mitophagy and mitochondrial dynamics. Mitophagy is a form of selective autophagy in which damaged mitochondrial fragment is devoured and degraded, triggered by cues that mark mitochondrial damage, such as imbalance in mitochondrial fission–fusion dynamics. ① As a fusion protein, MFN2 can be phosphorylated by Tensin homologue (PTEN)‐induced putative kinase 1 (PINK1). ② Parkin is recruited to OMM to phosphorylate the OMM protein and recruit and activate the E3‐ubiquitin ligase Parkin (PRKN). ③ Activated Parkin ubiquitinates different targets, such as LC3. ④ The damaged mitochondria are disintegrated by the lysosomes fusing with autophagosomes to form the so‐called “autolysosomes.” ⑤ Activated Parkin polyubiquitinates proteins on OMM, including MFN1/2, which leads to fragmentation ⑥ BCL‐2/adenovirus E1B interacting protein 3 (BNIP3) and FUN14 domain containing 1 (FUNDC1) on OMM interact directly with LC3 to promote mitophagy, which can be regulated by OPA1 and DRP1.

Mitophagy is a form of selective autophagy in which damaged and dysfunctional mitochondrial fragment are devoured and degraded, triggered by cues that mark mitochondrial damage, such as imbalance in mitochondrial fission–fusion dynamics.^84^ Mitophagy is promoted via specific OMM receptors, such as BNIP3, BNIP3L and FUNDC1 or ubiquitin molecules conjugated to proteins on the mitochondrial surface resulting in the formation of autophagosomes surrounding mitochondria and ultimately mitochondrial degradation (Figure 2). Mitophagy‐mediated elimination of mitochondria plays an important role in mitochondrial quality control and is closely related to cell fate such as senescence,^85^ apoptosis, and necroptosis.^86^ Defects in mitophagy have been linked to multiple diseases, including CVD, neurodegenerative disorders, cancer and lung disease. The cross‐action of some proteins in mitochondrial dynamics and mitochondrial autophagy pathways suggests that mitochondrial dynamics and mitochondrial autophagy are mutually influencing and interdependent.

Multiple signaling pathways have been implicated in mitophagy. Tensin homologue (PTEN)‐induced putative kinase 1 (PINK1), a serine/threonine kinase in healthy mitochondria, is continuously imported to the IMM, cleaved, and degraded by the mitochondrion‐specific proteases PARL and mitochondrial processing peptidases.^87^ When specific mitochondrial protein that cleaves PINK1 is knocked out, misfolded membrane protein is encountered or MMP becomes abnormal, resulting in the aggregation and stabilization of PINK1 on the OMM and in turn inducing the kinase domain of PINK1 to phosphorylate the OMM protein and recruit and activate the E3‐ubiquitin ligase Parkin (PRKN). Parkin is activated to polyubiquitinate proteins on OMM, such as MFN1/2, voltage‐dependent‐anion channel 1, and mitochondrial rho 1, for subsequent phosphorylation by PINK1. Phosphorylation of MFN2 as a Parkin receptor facilitates Parkin recruitment to the OMMs, and ultimately phosphorylates Parkin. Once activated, Parkin ubiquitinates different targets, such as LC3. Then the interaction of LC3 with the mitophagy adaptors mediates the endocytosis of impaired mitochondria into autophagosomes. Finally, the damaged mitochondria are disintegrated by hydrolase in the lysosomes, which fuse with autophagosomes to form the so‐called ‘autolysosomes’ (Figure 2). In addition, the ubiquitination of MFN1 and MFN2 leads to mitochondria fission, fragmentation, and subsequent mitophagy degradation duo to the formation of autophagosomes.^88^, ^89^

Other Parkin‐independent mechanisms involve Bcl‐2 homology 3‐only protein NIX and its homologue BCL‐2/adenovirus E1B interacting protein 3 (BNIP3), which interact directly with LC3 as a mitophagy receptor. NIX/BNIP3 mediates mitophagy through hypoxia and the stabilization of HIF‐1α.^90^ Another OMM protein, FUN14 domain containing 1 (FUNDC1), is a mitophagy receptor for hypoxia‐induced mitophagy.^48^ Phosphoglycerate mutase 5 dephosphorylates FUNDC1, promotes its interaction with LC3, and induces mitophagy under hypoxic stimulation. ULK1 is essential for inducing autophagy and interacts with FUNDC1 to phosphorylate at serine 17, thereby enhancing the binding of FUNDC1 to LC3 in mitochondria.^91^, ^92^, ^93^ Furthermore, FUNDC1 interacts with OPA1 and DRP1, which is regulated by serine 13 phosphorylation state during hypoxia or MMP loss.^94^ P62/SQSTM1, the first described mammalian selective autophagy receptor, is a cytoprotein containing a cytosolic LIR motif protein, and the LIR motif of P62 interacts with multiple sites on LC3.^95^ Except for receptor proteins described above, other cytoplasmic receptor proteins, for example NBR1, NDP52, and optineurin mediate mitophagy.^96^, ^97^ In addition, the OMM protein FK506 binding protein FKBP38 (also known as KFBP8) can induce Parkin‐independent mitophagy through the LIR motif aggregation of the LC3A.^98^

Mitochondrial fusion and fission are accompanied by mitophagy (Figure 2). Given that the sizes of autophagosomes in mammalian cells are approximately 500−1500 nm,^99^ predicting that a large network of mitochondria should be broken up by fission before mitophagy occurs is reasonable. In this regard, the degradation of mitochondrial fusion– or fission–related factors is required for mitophagy. First, mitochondrial fusion protein has a great effect on mitophagy. Decreased OPA1 leads to reduced mitochondrial fusion, which contributes to separate dysfunctional mitochondria, making them easy to be detected and removed by mitophagy.^67^ The interaction of BNIP3 with OPA1 induces mitochondrial fragmentation. MFN2 can be phosphorylated by PINK1, thereby promoting the recruitment of Parkin to OMM and inducing Parkin‐dependent mitophagy.^100^ As a fusion protein, the involvement of MFN2 in mitophagy is a further element that suggests reciprocity between mitochondrial dynamics and mitophagy.^22^ Moreover, mitochondrial fission is essential for mitophagy. DRP1 is a mitochondrial fission protein that interacts with overexpressed FUNDC1 and BCL2L13 to facilitate mitochondrial fragmentation and Parkin‐mediated mitophagy.^101^ Interestingly, DRP1‐mediated fission is proved to counteract aspecific Parkin phosphorylation, preventing undamaged mitochondria from mistargeted and unnecessary PINK1‐Parkin dependent mitophagy. DRP1‐mediated mitochondrial fission enhanced autophagy, while inhibition of DRP1 weakened BNIP3‐mediated autophagy. Another study reported that FIS1 overexpression increased mitochondrial fragmentation, leading to mitochondrial dysfunction and the increasement of autophagosome formation.^102^ Additionally, TBC1D15 interacts with FIS1 to mediate the autophagic encapsulation of mitochondria downstream of Parkin activation.^103^ In summary, mitophagy is functionally related to mitochondrial dynamics through interactions between mitochondrial dynamic factors and mitophagy receptors. Mitochondrial dynamics is closely coordinated with the mitophagy pathway by either promoting the fission–induced detachment of an impaired fragment or re‐fusing the damaged mitochondria to the normal mitochondrial network to weaken the influence of dysfunction.

## 3 MITOCHONDRIAL DYNAMICS AND DISEASE

Fusion, fission, and mitophagy are essential for normal mitochondrial functioning, energy metabolism, and cell fating. Inadequate or excessive levels of these processes upset the balance needed for proper mitochondrial function, ultimately resulting in all types of cell damage, especially energy‐intensive cells, including cardiomyocyte, neurons, tumor cells, and lung epithelial cells, which are particularly vulnerable to mitochondrial dysfunction in clinical evidence.^57^

### 3.1 Mitochondrial aberrations in cardiovascular conditions

#### 3.1.1 Key components aberrations in CVDs

CVDs remain the leading causes of death worldwide in recent years, accompanied by immense economic and health burdens. The high energy requirement of the heart requires adaptive mitochondrial dynamics to maintain normal function, which is emerging as a core player in cardiovascular homeostasis. Although the description of mitochondrial dynamics in patients with CVDs is limited because of inadequate detection methods, increasing lines of evidence suggest that impairment in mitochondrial dynamics contributes to various cardiovascular pathologies, including cardiac hypertrophy, HF, ischemia–reperfusion (IR) injury, and metabolic and genetic cardiomyopathy (Table 1).^22^, ^104^, ^105^

**TABLE 1:** The role of mitochondrial dynamics and mitophagy in cardiovascular disease and target treatment.

Disease	Mitochondrial dynamics	Mitochondrial morphology	Property	Treatment and outcome
HF	DRP1, FIS1, and BNIP3 are upregulated^18^, ^107^, ^108^	Excessive fission, fragmentation	Exacerbate	Sacubitril/Valsartan (LCZ696) and Mdivi‐1 as DRP1 inhibitor improve heart function in cardiomyopathy and heart failure^128^
				Treatment with mdivi‐1 in pigs did not reduce MI size or preserve cardiac function^129^
HF	MFN2 is downregulated^70^	Excessive fission, fragmentation	Exacerbate	Szeto‐Schiller SS‐31 (also named elamipretide), normalized the increased levels of fission‐associated proteins and the decreased levels of the fusion‐associated ones^114^
HF	Cardiac deletion of YME1L^25^	Fragmented	Exacerbate	
HF	Improper OPA1 processinzz^25^	Fragmented	Exacerbate	Concomitant deletion of OMA1 improves mitochondrial morphology and cardiac function^25^
HF	OPA1 mutation^25^, ^114^	Insufficient mitochondrial fusion	Exacerbate	Elamipretide^114^
HF	OPA1 downregulation^25^, ^114^	Insufficient mitochondrial fusion	Exacerbate	Melatonin and Calenduloside E play a beneficial role in attenuating HF by increasing the expression of OPA1^115^, ^116^
HF	PINK1 reduced^118^, ^122^	Impaired mitophagy	Exacerbate	Tat‐Beclin 1, a potent inducer of autophagy attenuated mitochondrial dysfunction and heart failure^64^
				Metformin prevents the progression of HF via activation of AMPK and PGC1α^70^, ^127^
HF	MFN1 phosphorylation		Exacerbate	A designed peptide (SAMβA) restores HF by MFN1–βIIPKC interaction^124^, ^125^
				Concomitant deletion of MFN1 in MFF‐knockout mice rescues mitochondrial and cardiac function; restored lifespan defects^126^
Cardiomyopathy	Improper OPA1 processing^25^		Exacerbate	
Cardiomyopathy	In Drosophila, cardiac gene silencing of MARF (analogue of the human MFN) and OPA1^113^		Exacerbate	Overexpression of MFN1 or MFN2 rescues cardiomyopathy^113^
Cardiomyopathy	Prolonged reduction of MFN2^12^, ^119^	Lack of fusion	Exacerbate	
Cardiomyopathy	The loss of MFN2 in the short term^12^, ^111^		Increased cellular proliferation, protective in cardiomyopathy	
Dilated cardiomyopathy	Cardiac MFN2 ablation^112^		Exacerbate	MItoQ reduces doxorubicin‐induced cardiomyopathy^123^
Dilated cardiomyopathy	Cardiac deletion of YME1L^25^	Fragmented	Exacerbate	
Idiopathic dilated cardiomyopathy	MFN1 decreased in patients^136^	Increased mitochondrial fragmentation	Exacerbate	Overexpression of MFN1 or MFN2 rescues cardiomyopathy^113^
Dilated cardiomyopathy	MTFP1 deletion^56^	Increased mitochondrial fragmentation, sensitizes mitochondria to mPTP opening	Exacerbate	
Cardiac hypertrophy	DRP1 upregulation and rapidly phosphorylated^109^, ^110^	Excessive fission	Exacerbate	Mdivi‐1 reduced cardiac hypertrophy^110^
Cardiac hypertrophy	Heterozygous deletion of DRP1^64^	Inhibition of mitophagy	Exacerbate	
Cardiac hypertrophy	Loss of PINK1 in mice^135^	Inhibition of mitophagy	Exacerbate	
I‐R	DRP1 upregulation and activated^104^, ^132^, ^133^, ^134^	Excessive mitochondrial fission	Exacerbate	P110,^134^, ^139^ mdivi‐1,^132^ and Pim1^140^ reduce I‐R by inhibiting DRP1
I‐R	DRP1‐knockout^18^		Exacerbate	
I‐R	DRP1 phosphorylation at serine 616 by RIP1^118^	Damaged mitochondria degradation	Protects the heart against ischemia	
I‐R	MFN2 deficient^131^	Reduced autophagy	Exacerbate	
I‐R	Cardiac MFN1 and MFN2 deletion^100^, ^119^, ^120^		Reduced cell death/increased survival	
I‐R	OPA1 downregulated^70^, ^114^, ^115^, ^116^		Exacerbate	Melatonin and Calenduloside E play a beneficial role in attenuating I/R by increasing the expression of OPA1^115^, ^116^
Abdominal aortic aneurysm (AAA)	DRP1 increased^117^	Promoted mitochondrial fission	Exacerbate	Mdivi‐1 protects AAA^138^
Vascular inflammation	DRP1 increased^38^	Promoted mitochondrial fission	Exacerbate	Macrophage‐specific Drp1‐knockout improves vascular inflammation^137^

Abbreviations: AAA, abdominal aortic aneurysm; DRP1, dynamin‐related protein 1; FIS1, fission protein 1; HF, heart failure; I‐R, ischemia–reperfusion; MARF, human analogue of MFN; Mdivi‐1, mitochondrial division inhibitor‐1; MFF, mitochondrial fission factor; MFN1, mitofusin1; MFN2, mitofusin2; MitoQ, mitochondria‐targeted antioxidants; mPTP, mitochondrial permeability transition pore; MTFP1, mitochondrial fission process 1; OMA1, mitochondrial metalloendopeptidase; OPA1, optic atrophy protein 1; P110, Drp1 inhibitor; Pim1, serine/threonine kinase; PINK1, PTEN‐induced putative kinase‐1; RIP1, receptor interacting protein‐1; YME1L, ATP‐dependent zinc metalloprotease.

In patients with HF, mitochondrial fragmentation, vacuolar degeneration with decreased mitochondrial size, and crista destruction are detected through transmission electron microscopy,^22^, ^70^, ^106^ along with the increased expression levels of DRP1, FIS1, and BNIP3 (Table 1).^18^, ^107^, ^108^ DRP1 is essential for cardiac embryonic development, confirmed by DRP1 deletion resulting in lethal heart defects.^22^ Furthermore, the expression of DRP1 increases in cardiac hypertrophy,^109^, ^110^ the levels of MFN1 and MFN2 decrease in patients with idiopathic dilated cardiomyopathy^111^, ^112^, ^113^ and HF,^70^ respectively, and these effects are correlated with increased mitochondrial fragmentation in the heart. Significant downregulation of OPA1 expression is found in HF^25^, ^114^ and cardiac IR injury.^70^, ^114^, ^115^, ^116^ OPA1 mutation and improper OPA1 processing induce mitochondrial fragmentation, resulting in severe cardiomyopathy^25^ and ultimately HF.^25^, ^114^ The roles of DRP1 in vascular endothelial cells and macrophages have been explored and found to be closely related with abdominal aortic aneurysm (AAA)^117^ and vascular inflammation.^38^ In 2022, a new and dispensable IMM protein MTFP1/MTP18 for mitochondrial division was indicated to be essential for cardiac structure and function in fatal and adult‐onset dilated cardiomyopathy.^56^ These data suggest that excessive mitochondrial division and insufficient mitochondrial fusion are associated with cardiac pathology. However, in contrast to these findings, DRP1‐knockout exacerbates myocardial IR injury,^18^ but DRP1 phosphorylation at serine 616 by RIP1 alleviates ischaemia.^118^ Moreover, the activation of DRP1 not only plays a regulatory role in myocardial fibrosis and myocardial hypertrophy as an adaptive response to exercise but also produces pathological effects during long‐term activation, aggravating myocardial fibrosis and myocardial hypertrophy.^7^ The lack of MFN2 in the short‐term can be protective by increasing cellular proliferation,^12^, ^119^ and prolonged decrease in MFN2 may result in serious cardiomyopathy defects due to a loss of fusion.^12^, ^111^ The single cardiac deletion of MFN1 may maintain cardiac function, and cardiac MFN2 deletion improves cardiac recovery following IR injury.^100^, ^119^, ^120^ These findings suggest that the role of mitochondrial dynamics in heart diseases cannot be generalized, is complex, and responds to various types of stress and cardiac disease conditions, involving the coordination of a variety of regulatory proteins. Notably, DRP1–MFN1–MFN2 cardiac triple‐knockout mice showed delayed cardiomyopathy development and long survival times,^7^, ^121^ whereas cardiac‐specific double MFN1 and MFN2 knockout^7^ or Drp1‐knockout^7^ mice developed rapid HF and premature death. It thus appears that disproportionate fission or fusion is more detrimental than simultaneous disturbance of two processes in the onset and development of CVDs.^7^

#### 3.1.2 Abnormal mitophagy in CVDs

In recent years, considerable attention has been drawn to the roles of mitophagy in cardiovascular disorders. Mitophagy dysfunction has been observed in various CVDs, but different mitophagy pathways play different roles. PINK–PRKN mitochondrial autophagy has a “double‐edged sword” role depending on the mode of activation in atherosclerosis and cardiomyopathy.^16^ Mitophagy plays a protective role in cardiomyocyte recovery after myocardial infarction (MI), HF progression, myocardial IR injury, and atherosclerosis by promoting the phagocytosis of damaged mitochondria, reducing ROS, and inhibiting apoptosis.^70^, ^141^, ^142^ Therefore, the activation of autophagy is a potential strategy for treating these diseases. The mild activation of PINK1–PRKN mitophagy in cardiomyocytes prevents HF development, alleviates MI insult, attenuates dilated cardiomyopathy,^16^, ^143^, ^144^ induces mitophagy by modulating the Mir302a‐3p–FOXO3 axis, and alleviates myocardial IR injury by inhibiting cardiomyocyte apoptosis and ROS.^16^, ^145^ Contradictory to these results, PINK1–PRKN mitophagy in vascular smooth muscle cells promotes atherosclerosis.^16^, ^17^ BNIP3‐mediated mitophagy potentiates HF,^16^ and aldehyde dehydrogenase 2 family member alleviates IR injury by inhibiting PINK1–PRKN mitophagy in a rat model and H9C2 cells under H/R.^146^ The ablation of AKAP1 by evoking mitophagy enhances pathological cardiac remodeling, infarct size, and mortality after MI.^141^, ^147^ Nuclear receptor subfamily 4 group A member 1 induces atherosclerosis by activating the CaMKII Parkin–mitophagy pathway,^141^, ^148^ and mitophagy is promoted duo to the development of dilated cardiomyopathy in MFF mutant mice.^126^, ^141^ Overall, the role of mitophagy differs according to damage in a CVD, and the mild activation of mitophagy prevents mitophagy dysfunction–initiated cardiac diseases.^149^ The constitutive activation of mitophagy may exert negative roles on cardiovascular cells, leading to abnormal responses, apoptosis, and deterioration of cardiac diseases.^16^ Mitophagy dysfunction is an attractive target for CVD treatment, but which depends on clear mechanism studies. Current studies have indicated that the mitophagy mechanism of cardiomyocytes is mainly controlled by PTEN‐induced putative kinase‐1 and PRKN,^16^, ^70^, ^150^ OMM, BNIP3, and FUNDC1 also take part in the mitochondrial autophagy mechanism.^70^

#### 3.1.3 Targeting mitochondrial dynamics therapy in CVDs

Most CVDs are characterized by abnormalities in multiple regulatory proteins in mitochondrial dynamics, such as DRP1, FIS1, MFN1, MFN2, OPA1, and Parkin, and targeting these proteins can be an effective approach for potential treatment.^7^, ^70^ However, whether constitutive or excessive activation or other mitophagy mediators are responsible for the adverse effects of mitophagy is unclear. Thus, therapeutic approaches involving mitophagy activation should be used in caution for the prevention of excessive mitophagy activation resulting in disease aggravation rather than CVD alleviation.^16^

As a specific DRP1 inhibitor, the quinazolinone derivative Mdivi‐1 attenuates cardiac dysfunction in diverse mouse cardiomyopathy models.^110^, ^128^, ^132^ likely because of mitochondrial elongation or reduced autophagy (Table 1). In addition, Mdivi‐1 has pleiotropic effects such as cleaving L‐OPA1 and altering the expression of OXPHOS complex proteins, ultimately increasing superoxide production.^151^ On the contrary, treatment of MI in pigs with mdivi‐1 did not reduce MI size or preserve cardiac function.^129^ P110, a small peptide specifically blocking the interaction between DRP1 and FIS1, improves cardiac structure and attenuates cardiac dysfunction in rat heart after IR injury.^134^, ^139^ SAMβA, a novel small peptide, improved mitochondrial and cardiac function by inhibiting the interaction of MFN1 with βIIPKC in a rat model for HF.^124^, ^125^ The small‐molecule S3 can stabilize mitochondrial fusion in adult cardiomyocytes.^152^ Metformin, as a first‐line treatment for diabetes, prevents the progression of HF and improves left ventricular function by activating AMPK and PGC1α in the mitochondrial dynamics regulation pathway.^70^, ^127^ Other drugs, such as calenduloside E and melatonin, effectively modulate OPA1 to improve cardiac function (Table 1).^115^, ^116^ In addition, melatonin and liraglutide reduce cardiac fibrosis, inflammatory responses, and myocardial death by promoting mitophagy.^116^, ^153^, ^154^, ^155^ Valsartan inhibits Parkin activity or mitophagy, ultimately alleviating left ventricular hypertrophy and increasing mitochondrial biogenesis in experimental hypertension.^156^ Cardiac‐specific knockout of MFN1 or MFN2 is associated with reduced cell death and improved survival during IR injury.^112^, ^120^ But, there has been reported that overexpression of MFN1 or MFN2 rescues cardiomyopathy.^113^

### 3.2 Mitochondrial dysfunction in neurodegenerative diseases

Neurodegenerative diseases constitute a spectrum of complex heterogeneous disorders characterized by the progressive death of nerve cells and loss of brain tissues,^157^, ^158^ and their treatments are limited. Given that nerve cells require high levels of mitochondrial metabolism for their functions, mitochondrial dysfunction has emerged as one of the predominant phenotypes in neurodegenerative diseases.^12^, ^69^, ^159^, ^160^ Excessive mitochondrial fission, downregulation of mitochondrial fusion, as well as a reduction in mitophagy, are observed in neurodegenerative disorders with diverse genetic and environmental causes supported by several lines of clinical evidence,^12^ including Charcot–Marie–Tooth disease (CMT) type 2A (CMT2A),^161^, ^162^ dominant optic atrophy (DOA),^163^ Parkinson's disease (PD),^164^ Huntington's diseases (HD),^165^ Alzheimer's disease (AD),^166^ and amyotrophic lateral sclerosis (ALS),^167^ which have various pathophysiological etiology.^168^ CMT2A, DOA, PD, and some intractable epilepsy are directly caused by mutations in dynamin‐related genes that are associated with mitochondrial fusion, fission, and mitophagy. For example, classical CMT2A and DOA are caused by heterozygous mutations in mitochondrial fusion‐related genes *MFN2* ^162^, ^169^, ^170^ and OPA1,^171^, ^172^ respectively. Two forms of inherited early‐onset PD are caused by mutations in serine/threonine kinase PRKN and Pink1.^48^, ^173^ AD and PD are the most common neurodegenerative disorders, which are discussed in detail next.

#### 3.2.1 Alzheimer's disease

AD is an aging‐related neurodegenerative disorder, which is clinically characterized by progressive memory loss, learning disabilities, and damaged cognitive function.^174^, ^175^ The intracellular aggregation of hyperphosphorylated tau protein and extracellular senile plaques composed of amyloid β (Aβ) deposits are two pathological hallmarks in AD brain, and mitochondrial dysfunction in neurons is a major hallmark of AD.^176^, ^177^ At present, AD is the major public health concerns worldwide, affecting 10% of people aged 65–75 years and about 32% of people over 80 years,^178^ and AD patients worldwide can reach 131 million by 2050.^179^ Although the intricacy of the mechanism of AD pathogenesis remains unclear, cumulative evidence indicates that impaired mitochondria likely play critical roles in the pathogenesis of AD.

Indeed, in AD patients and model organisms mitochondrial fragmentation was confirmed. Excessive mitochondrial fission leads to compromised neuronal function caused by decreased energy production by interfering with OXPHOS complex assembly and cristae integrity.^69^ Moreover, as a precursor to cell apoptosis, fission may directly cause neuronal death. Correspondingly, biochemical evaluation of AD brains demonstrated significantly increased DRP1 and FIS1, and reduced MFN1, MFN2, and OPA1 mRNA and protein levels.^69^, ^180^, ^181^ Despite some controversial reports about the DRP1 levels, significant changes in the posttranslational modifications of DRP1 were reported by several groups. S‐nitrosylation of DRP1 induced by Aβ triggered mitochondrial fission and neuronal damage in AD brain.^182^ Significantly increased DRP1 phosphorylation at the Ser616 site increased mitochondrial DRP1 in AD brain or stimulated DRP1 translocation to the mitochondria and fission activity.^183^

On the contrary, mitochondrial fusion/fission imbalance may affect the distribution and quantity of mitochondria in synapses, resulting in impaired synaptic excitation transmission. In addition, axonal transport inhibition is closely associated with unbalanced fission/fusion by increasing microtubule instability, including anterograde transport and retrograde transport.^184^, ^185^ Deletions or mutations in MFN2 can slow down axonal transport in both directions.^186^ A recent study has shown that MFN2 participates in mitochondrial transport by interacting with mitochondrial Rho small GTPase.^187^ In AD, impaired axonal transport precedes the aggregation of mitochondrial fragment and toxic protein compounds and is correlates with disturbed synaptic function.^185^, ^188^ However, the precise underlying molecular mechanisms of how unbalanced fission/fusion affects axonal transport remains to be defined. Moreover, the progression of AD has been associated with reduced mitophagy and cristae disorders.^189^, ^190^

#### 3.2.2 Parkinson's disease

PD is the second most common neurodegenerative disorder after AD, and PD is considered as a multifactorial disease. Aging has been identified as a great risk factor for the initiation and progression of PD,^191^ while genetic disorder and environmental toxicity are thought to increase the risk for PD.^192^, ^193^ Familial PD is widely believed to be duo to genetic mutations, whereas the cause of idiopathic PD remains unknown.^79^ To date, more than 20 genes and some environmental factors have been identified to cause PD, which also influence various mitochondrial aspects, including dynamic changes (fusion, fission), mitophagy, Ca^2+^ homeostasis, oxidative stress, and mitochondrial biogenesis.^194^, ^195^, ^196^ These findings indicate that mitochondrial dysfunction plays a central role in PD pathogenesis.

All five major human genes involved in PD have critical functions in mitochondrial regulation, such as PRKN, PINK1, deglycase DJ1, single‐nucleotide polymorphism rs356219 in α‐syn (SNCA), and leucine‐rich repeat kinase 2 (LRRK2).^197^ For example, in neuroblastoma cells, the α‐Syn protein encoded by the over‐expression of SNCA increases DRP1 and facilitates its translocation into the mitochondria, thereby modifying mitochondrial fission.^198^ Endogenous LRRK2 in neurons directly interacts with and phosphorylate DRP1 to elevate mitochondrial DRP1 levels, thereby increasing the mitochondrial fragmentation.^199^ The LRRK2 G2019S mutant in PD was found to induce excessive mitochondrial fragment in HEK293T cells.^200^ Similarly, DJ‐1 regulates the mitochondrial dynamics by modulating the expression of DRP1 and DRP1‐dependent fission to protect neurons against oxidative stress.^201^, ^202^ In addition, PINK1 and PRKN are closely related to mitochondrial dynamics, although they are often involved in regulating mitochondrial quality control as master adaptors for mitophagy.^203^ Notably, the relationship between PINK1/Parkin and mitochondrial fission is unclear and such a relationship may vary depending on the cell and species context. For instance, in murine M17 dopaminergic neurons, *Pink1* silencing increases mitochondrial fission.^204^ On the contrary, in rat dopaminergic cells, *Pink1* shRNA silencing elongated mitochondria by suppressing Drp1 S616 phosphorylation.^205^ PINK1 also has a neuroprotective function by promoting MFN2‐mediated mitochondria fusion, as demonstrated in DA neuron cells where PINK1 dysfunction inhibits MFN2 expression causing cells to come more sensitive to neurotoxins.^204^ Interestingly, MFN2, rather than MFN1, is crucial for the axon projection of dopamine (DA) neurons in midbrain. MFN2 knockout causes a reduction in DA nerve terminals and DA levels in striatum, but has no impact on the number of DA neurons in the midbrain.^19^, ^206^ MFN2 overexpression protects DA neurons from neurotoxicity and mitochondrial dysfunction induced by paraquat.^207^ These results suggest that MFN2 plays a complex role in PD neuronal protection.

Furthermore, some environmental factors associated with idiopathic PD pathogenesis are also mitochondrial toxins,^208^ such as 6‐hydroxy dopamine (6‐OHDA), 1‐methyl‐4‐phenyl‐1,2,3,6‐tetrahydropyridine (MPTP), rotenone, and paraquat.^209^, ^210^, ^211^ These toxins inhibit the mitochondrial electron transport chain (ETC), which results in mtROS production, ATP depletion, and defective mitochondrial biogenesis/dynamics, thereby increasing mitophagy as well as triggering apoptosis and the death of dopaminergic neurons.^212^, ^213^ Rotenone also disrupted the ETC and heightened caspase‐1 cleavage, resulting in an increase of the NLRP3 inflammasome in bone marrow‐derived macrophages.^208^

Therefore, mitochondrial dysfunction caused by abnormal mitochondrial dynamics plays a key role in the pathogenesis of AD and PD. Interestingly, inherited mitochondriopathies do not usually exhibit the characteristics of PD.^214^ Moreover, in ATP13A2 mutation carriers with Kufor‐Rakeb syndrome, mitochondrial dysfunction is the consequence rather than the cause of disease pathophysiology.^215^, ^216^, ^217^ So, whether these defects are causative or secondary to another pathogenic process remain challenging.^164^

#### 3.2.3 Targeting mitochondrial dynamics for neurodegenerative diseases

Overwhelming studies indicate dysfunctional mitochondrial fusion–fission dynamics contributes to the onset and development of neurodegenerative disorders (Table 2). Thus, modulating mitochondrial fusion and fission with either genetic approaches or small molecules in neurodegenerative disease models improves function.^12^ For example, adeno‐associated virus (AAV) injection of WT OPA1 alleviates retinal ganglion cell degeneration in mouse DOA models.^218^ Similarly, the injection of neuronal AAV carrying a dominant negative variant of DRP1 successfully reduces DA neuron degeneration in environmental and genetic PD mouse models.^219^ Crossing DRP1^+/−^ mice with genetic mouse models of AD reduces DRP1 levels and alleviates mitochondrial dysfunction and neurodegeneration.^220^, ^221^ Meanwhile, reducing DRP1 recruitment to mitochondria by inhibiting DRP1 phosphorylation exerted a protective effect against neurodegeneration in an AD model.^183^, ^222^ These data underline that aberrant mitochondrial structure and function contribute to multiple types of neuronal dysfunctions and forecast that reducing DRP1 expression or activity are appealing therapeutic approaches. Cumulative evidence indicates that Mdivi‐1 is a promising therapeutic that attenuates neuronal cell death through mitochondrial elongation, enhances resistance to apoptosis, inhibited complex I activity and ROS generation in neurodegenerative disease and neurotoxicity models, such as PD,^223^, ^224^ AD,^183^, ^225^ and pediatric anesthesia.^12^, ^226^, ^227^, ^228^ P110 improves function in many models of neurodegenerative diseases, such as PD, AD, HD, and ALS (Table 2).^229^, ^230^, ^231^, ^232^ Notably P110 had no observable effects on a wild‐type mouse compared with an ALS model mouse, indicating that it may selectively inhibit pathological division while maintaining normal physiological division activity.^230^ Some small molecules, such as chimera B‐A/I, MP1Gly, MP2Gly, and S3, treat a variety of neurodegenerative diseases by altering mitochondrial fusion activity (Table 2).^233^, ^234^ Clinical trials have shown that as a partial inhibitor mitochondrial complex, metformin provides protection against cognitive decline in patients with AD, but its molecular targets and mechanism need further study.^235^ Mitochondrion‐targeted antioxidants CoQ10 and MitoQ have shown positive outcomes in the animal models of PD and mitochondrial dysfunction.^236^ In addition, isolated mitochondria were delivered systemically in a PD model mouse and found to improve behavioral deficiencies.^237^

**TABLE 2:** Therapeutics targeting mitochondrial dynamics in neurodegenerative disorders.

Disease model or disease	Dysfunctional mitochondrial dynamics	Treatment	Outcome	References
Mouse dominant optic atrophy (DOA) model	Fragmented	Adeno‐associated virus (AAV) injection of WT OPA1	Attenuated loss of retinal ganglion cells (RGCs)	^218^
Both genetic and environmental PD mouse models	Fragmented	Injection of a dominant negative variant of DRP1 by neuronal AAV	Reduced dopaminergic neuron degeneration	^219^
Cultured fibroblasts and neurons defected	Mitochondrial fusion defects	TAT‐367‐384Gly	Stimulated mitofusins	^234^
Cultured fibroblasts and neurons defected CMT3A‐associated genetic	Mitochondrial fusion defects	TAT‐398‐418Gly	Inhibited mitofusins and aggravated mitochondrial dysmorphology	^234^
Mouse models of PD and AD	Fragmented	Mdivi‐1	Improved behavior outcomes and reduced neurodegeneration	^183^, ^223^, ^224^, ^225^
Mouse models for PD, AD, multiple sclerosis, and ALS	Excessive mitochondrial fission	P110	Rescued mitochondrial function and cognitive defects	^229^, ^230^, ^231^, ^232^
Cell culture model of CMT2A	Inhibited mitochondrial fusion and subcellular trafficking	Chimera B‐A/I	Restored mitochondrial morphology	^233^
Parkinson disease	Compromise mitophagy	MitoQ	Improved mitochondrial dynamics and behavior outcomes	^236^
Mouse model of Parkinson's disease induced by respiratory chain inhibitor MPTP	Impaired ETC	Isolated mitochondria delivered systemically	Increase behavioral outcomes	^237^
AD patients	Fusion/fission imbalance	Metformin	Protects against cognitive decline	^235^
Models of AD	Fragmented	Urolithin A and Actinonin	Reverses memory impairment	^238^

Abbreviations: AD, Alzheimer's disease; ALS, amyotrophic lateral sclerosis; Chimera B‐A/I, a small molecule; CMT2A, Charcot–Marie–Tooth syndrome type 2A; CMT3A, Charcot–Marie–Tooth Syndrome Type 3A; Mdivi‐1, mitochondrial division inhibitor‐1; Mitofusin agonist; MitoQ, mitochondria‐targeted antioxidants; OPA1, optic atrophy protein 1; P110, Drp1 inhibitor; PD, Parkinson's disease.

#### 3.2.4 Mitophagy as double‐edged sword in neurodegenerative diseases

Aging of the nervous system combined with decreased mitophagy is a hallmark of neurodegeneration.^239^, ^240^ Mitophagy dysregulation emerges as a predominant factor contributing to various neurodegenerative disorders.^84^ The mitophagy pathway is affected at multiple stages, including cargo recognition, adaptor recruitment, and fusion of damaged mitochondria with lysosomes containing hydrolases. Thus, the roles of mitophagy in neurodegenerative disorders are complicated. For example, a defective mitophagy pathway is a dominant pathophysiology phenotype of AD, HD, ALS, and mixed dementia,^238^, ^241^, ^242^, ^243^, ^244^ and inducing mitophagy can result in pathological and cognitive outcomes in AD.^238^ However, suppressing mitophagy in some models restored mitochondrial density in synapses.^245^, ^246^ Parkin and PINK1 are important hidden risk factors for the early‐onset development of PD and HD, which are promoted by defective PINK1 and Parkin.^19^, ^247^ The importance of Parkin was demonstrated in Parkin‐knockout mice with mitochondrial abnormalities, motor deficits, and neuronal deficiency.^248^ Intriguingly, PINK1 or Parkin knockout mice failed to develop spontaneous PD, whereas PINK1‐deficient rats showed aggravated neurodegeneration and behavioral deficits in PD.^249^ However, Parkin‐deficient rats showed no abnormalities, indicating that abrogated PINK1 or Parkin contributes to PD but is not the only cause of PD. The Parkin‐independent role of PINK1 drives the phenotype.^240^ In ALS, mitophagy may exert neuroprotection in the initial stages, but prolonged elevated mitochondrial autophagy may negatively affect neuronal survival.^84^, ^246^

The pathogenesis of PD and AD may be a cycle and feedback loop in which nerve cell apoptosis leads to mitochondrial dysfunction, which can further aggravate cell apoptosis. Whether damage to mitochondria is a cause or an effect of neurodegeneration is unclear, and thus modifying mitochondrial dynamics or mitophagy is not the sole consideration or treatment.^84^, ^240^

### 3.3 Cancer and corresponding therapeutic approaches

The second leading cause of death globally is cancer,^250^ which is characterized by the infinite proliferation, metastasis, and recurrence and prone to drug resistance. These processes are inextricably connected with dysregulated mitochondrial refusion, fission, and mitophagy.^250^, ^251^ Mitochondria are heterogeneous organelles with morphology and function highly dependent on the surrounding environment. Therefore, the role of mitochondrial dynamic in tumors is complex and cannot be generalized. The distinct roles of mitochondrial fission, fusion, and mitophagy have been elucidated in different cancer contexts (Table 3). In most cases, mitochondrial fission facilitates the cancer cell proliferation, migration, and drug resistance, causing cancer development.^252^, ^253^ Excessive mitochondrial fission plays a causal role in promoting cancer cell transformation.^254^ Many tumors exhibit fragmented mitochondria with upregulated DRP1 and decreased expression levels of MFN1 and MFN2^255^ in ovarian cancer,^256^ HCC,^257^ lung cancer,^258^ colon cancer,^259^ breast cancer,^252^ neuroblastoma,^260^ and glioblastoma,^253^ which are correlated to the metastatic potential of cancer cells. Increased DRP1 or DRP1‐Ser616 phosphorylation levels can prevent apoptosis, change cellular metabolism, induce immune escape, sustain cell cycle and proliferation in tumor cells, and ultimately promote the occurrence and development of tumors.^10^, ^261^, ^262^, ^263^ Thus, DRP1 inhibition may decrease tumor cell proliferation and invasion and is supported in several tumor models treatment with DRP1 inhibitor, such as Mdivi‐1, vemurafenib, Drpitor1, chloroquine, and isorhamnetin (Table 3),^252^, ^264^, ^265^, ^266^, ^267^ indicating that DRP1 inhibition is a potential therapeutic approach for tumors. In some cancers, mitochondrial fusion supports tumor cell growth in NSCLC, HCC, breast and cervical cancer, and other tumors.^257^, ^268^, ^269^, ^270^ OPA1 and MFN1 are upregulated in ovarian, breast, liver cancer, AML, esophageal, renal, and stomach cancer (Table 3), and another RNAseq data have also stressed the significant mRNA overexpression of OPA1 in a variety of tumor types, including breast, renal, stomach, and esophageal cancer. Meanwhile, the upregulation of DRP1 is associated with drug resistance in a variety of tumors, including ovarian cancer, NSCLC, and colorectal cancer (Table 3).^271^, ^272^, ^273^, ^274^, ^275^ Consistently, mitochondrial fission in ovarian cancer proteolytic processing of OPA1 in gynecologic tumors is associated with cisplatin chemoresistance.^273^, ^276^ However, mitochondrial fission inhibition induced by DRP1 phosphorylation on Ser637 leads to drug resistance in breast cancer.^10^, ^272^ The formation of a large fused mitochondrial network after the upregulation of *MFN1* and *MFN2* genes and OPA1‐mediated cristae morphological changes are associated with drug resistance.^124^, ^276^, ^277^ Furthermore, FIS1 phosphorylation promotes mitochondrial fission and HCC metastasis, and the expression level of FIS1 increases in leukemic stem cells (Table 3).^278^, ^279^ Indeed, mitochondrial fission plays an important role in CSC stemness, but the mechanisms involved remains elusive. Different tumor stem cells have different mitochondrial phenotypes and responses to mitochondrial dynamics involving regulatory proteins. DRP1 inhibition by Mdivi‐1 decreases YAP/TAZ‐dependent clonogenicity in human mammary epithelial cells.^268^ Coherently, DRP1 S616E S637A overexpression upregulates stemness gene expression in human glioblastoma PDX.^261^ MFF downregulation contributes to CSC exhaustion in prostate cancer and decreases cancer stem cell proliferation (Table 3).^280^ By contrast, DRP1 and MFN1/2 knockout decreased stem cell differentiation in mouse embryonic cortex development and human mammary epithelial cells, respectively, and OPA1 shRNA knockdown had the same effect on human breast cancer cell lines.^281^, ^282^ Thus, mitochondrial division and fusion are involved in the differentiation and self‐renewal of tumor stem cells. Notably, MFN2 plays roles in tumorigenesis and development. MFN2 upregulation promotes cell survival in HCC, breast cancer, and cervical cancer,^268^, ^270^ but has an opposite effect in HCC and lung cancer.^258^, ^283^ And MFN1 also showed different effects in different tumors (Table 3). Increased MFN2 level suppresses ovarian cancer and NSCLC progression by promoting autophagy and reducing ROS by downregulating AMPK/mTOR/ERK signaling.^269^, ^284^ As discussed above, mitochondrial fission and fusion are pleiotropic in cancer cells and have different functions depending on cell context.

**TABLE 3:** Summary of key regulators of mitochondrial dynamics and mitophagy in cancer.

Protein/gene	Disordered	Cancer/disease relevance	Outcome	Treatment
DRP1	Upregulation	Ovarian cancer,^256^ lung cancer,^258^ colon cancer,^259^ breast cancer,^252^ neuroblastoma,^260^ glioblastoma,^253^ melanoma,^262^ prostate cancer,^299^ pancreatic cancer,^300^ renal carcinoma,^301^ HCC^257^	Associated with metabolic reprogramming, cell cycle progression, and increased migration, invasiveness, and metastatic capacities	Mdivi‐1 decreased the proliferation of the lung and the colon cancer cells^264^ Combination of chloroquine (CQ) and isorhamnetin (IH) enhances intriple‐negative breast cancer (TNBC) cells apoptosis^265^ Mdivi 1 has off‐target effects on the ETC^266^ Silencing DRP1 inhibited the metastatic capacity of breast cancer cells^252^ Vemurafenib triggered death in melanoma cell models^267^
DRP1	Enhanced Drp1 phosphorylation	Lung cancer^298^	Suppressed cell migration and invasion	
DRP1	Upregulation or activation	Ovarian cancer, breast cancer, NSCLC, colorectal cancer^271^, ^272^, ^273^, ^274^, ^275^	Drug resistance	Mdivi‐1 restored cisplatin sensitivity and prevented disseminated cancer‐cell awakening in lung cancer^304^
DRP1	DRP1 phosphorylation on Ser637	Breast cancer^10^, ^272^	Tamoxifen resistance	
DRP1	Overexpression	Human glioblastoma^261^	Inhibits differentiation and upregulates stemness gene expression	Downregulation of Drp1 by shRNA‐DISC1 knockdown inhibits glioblastoma cell migration and invasion^307^ Drp1 shRNAs or midivi‐1 induced brain tumor initiating cells (BTICs) apoptosis and inhibited tumor growth^261^
DRP1	Depletion	Neural stem cell (NSC)^282^	Decreased differentiation potential and increased self‐renewal	Rapamycin rescues mitochondrial alterations in glioblastoma cells by inducing the expression of DRP1^305^ Autophagy activation associates with suppression of prion protein and improved mitochondrial status in glioblastoma cells^306^
MFF	Upregulation	Prostate cancer^280^	Increases cancer cells and stem cell proliferation	Knockdown of Mff caused CSC exhaustion and loss of tumorigenic capability^280^
FIS1	FIS1 phosphorylation	Hepatocellular carcinoma^279^	Facilitated metastasis	
FIS1	Upregulation	Leukemic stem cells (LSCs)^278^	Maintain the stemness	Depletion of FIS1 using shRNA strongly reduced the colony‐forming ability of primary AML cells^278^
MFN1	Downregulation	HCC, breast cancer^283^, ^297^	Cancer cell survival and metastasis	Protodioscin induces mitochondrial apoptosis of human hepatocellular accompanied with MFN1 upregulation^302^ Silibinin inhibits migration and invasion of breast cancer cells through upregulating the expression of OPA1, MFN1, and MFN2^303^ Melittin has anticancer effects on 4T1 cell lines by upregulation of Mfn1 and Drp1 mRNA expression^304^
MFN1	Knockdown	Normal mammary epithelial cells and in BT549 and MDA‐MB‐231 stem cell‐rich breast cancer cell lines^281^	Decreases stemness	
MFN1	Upregulation	Prostate cancer^316^	Cancer cell survival	CGP37157 (CGP), an inhibitor of mitochondrial calcium efflux, induces apoptosis in prostate cancer cells by MFN1 degradation^317^
MFN2	Downregulation	Breast cancer, lung adenocarcinoma^258^, ^283^	Cancer cell survival under metabolic stress	Leflunomide decreased the proliferation and metastasis of cancer cells in NSCLC and ovarian cancer by activating MFN2^269^, ^284^
MFN2	Upregulation	HCC, breast cancer, cervical cancer^268^, ^270^	Cell survival	
OPA1	Upregulation	Ovarian, breast, liver cancer, AML, esophageal, renal, and stomach cancer^315^	Increased cancer cell proliferation	Silencing of OPA1 could inhibit tumor growth in breast cancer experimental models^315^ Small‐molecule OPA1 inhibitor MLYS22 inhibited breast cancer cells proliferation^254^
OPA1	Upregulation	Ovarian cancer^273^, ^276^	Chemoresistance	
OPA1 or MFN1/2	Knockout	Neural stem cell (NSC)^282^	Maintain the stemness	
Parkin	Deleted or inactivating mutations	Ovarian, breast, lung and bladder cancers, glioblastoma, liver tumors^96^, ^285^, ^287^	Proliferation and metastasis	Combination of with generic autophagy inhibitor hydroxychloroquine with trametinib significantly reduced pancreatic ductal adenocarcinoma (PDAC) tumor burden^293^, ^294^ The overexpression of Parkin and PINK1 in breast and glioma cells attenuates cellular proliferation^289^, ^290^
PINK1	Reduced expression	Glioblastoma and ovarian cancer; neuroblastoma; pancreatic ductal adenocarcinoma^285^, ^287^, ^290^	Proliferation and metastasis	PINK1 overexpression attenuates in vivo glioblastoma growth in orthotopic mouse xenograft models and a transgenic glioblastoma model in Drosophila^290^, ^305^
PINK1 /Parkin	Increased	Head and neck squamous cell carcinoma, bone marrow‐derived mesenchymal stem cells^314^	Maintain the stemness	Melatonin and verteporfin synergistically suppress the stemness of head and neck squamous cell carcinoma through the downregulation of PINK1^295^ SiRNA Pink1 siRNA led to diminished stemness of the stem cells^314^
BNIP3	Deleted, silenced or mis‐localized	Breast, prostate, colon, pancreatic, liver, glioma and other cancers, breast cancer^311^, ^312^, ^313^	Proliferation and metastasis	AT‐101 lead to autophagic cell death^296^
BNIP3	Deleted, silenced or mis‐localized	PDAC^309^, ^310^	Drug resistant	Re‐expression of BNIP3 promoted drug sensitivity in PDAC^309^, ^310^

Abbreviations: AML, acute myelogenous leukemia; BINP3, Bcl‐2/E1B19kDa‐interacting protein; DRP1, dynamin‐related protein 1; ETC, electron transport chain; FIS1, fission protein 1; HCC, hepatocellular carcinoma; Mdivi‐1, mitochondrial division inhibitor‐1; MFF, mitochondrial fission factor; MFN1, mitofusin1; MFN2, mitofusin2; NSCLC, non‐small cell lung cancer; OPA1, optic atrophy protein 1; PDAC, pancreatic ductal adenocarcinoma; PINK1, PTEN‐induced putative kinase‐1.

Mitophagy is another important component of mitochondrial quality control, which is pleiotropic in cancer. First, as the accumulation of dysfunctional mitochondria is involved in oncogenesis, mitochondrial autophagy seems to be a tumor‐suppressive system that clears depolarized mitochondria and prevents Warburg metabolism and excess ROS production.^285^, ^286^ As shown in Table 3, the expression of Parkin is lost in various types of cancer, such as human breast, lung, ovarian, bladder cancer and other cancers.^96^, ^285^, ^287^ Inactivating mutations are observed in glioblastoma and other cancers,^288^ and the overexpression of Parkin and PINK1 in breast and glioma cells attenuates cellular proliferation.^289^, ^290^ Mitophagy promoted by another E3 ubiquitin ligase (ARIH1) protects cancer cells and causes drug resistance in response to chemotherapeutic agents.^251^ Given the autophagy role in drug resistance, the application of autophagy inhibitor in combination with other drug regimens is advancing in clinical trials for many types of human cancer.^291^, ^292^ For example, chloroquine (autophagy inhibitor) in combination with trametinib shows significant efficacy in reducing tumor burden in pancreatic ductal adenocarcinoma (Table 3),^293^, ^294^ although to what extent these effects are mediated through the inhibition of general autophagy versus inhibition of mitophagy functions is unclear. Melatonin and verteporfin synergistically reduce the growth of head and neck squamous cell carcinoma by decreasing Parkin and PINK expression.^295^ However, BNIP3/BNIP3L‐dependent mitochondrial autophagy induced by cotton seed‐derived compound AT‐101 leads to autophagic cell death in apoptosis lacking tumor cells.^296^

In summary, therapeutic interventions targeting mitochondrial fission, fusion, or mitophagy hold promise in the treatment or management of cancers, but more studies are necessary because of mitochondrial heterogeneity.

### 3.4 Pulmonary disease and corresponding therapeutic approaches

Pulmonary diseases are among the most common diseases worldwide. A variety of factors, such as smoking, bacteria, viruses, air pollution, and genetic factors, largely contribute to the development of these diseases. Common pulmonary diseases include acute lung injury (ALI)/acute respiratory distress syndrome (ARDS), chronic obstructive pulmonary disease (COPD), asthma, pulmonary fibrosis (PF), pulmonary arterial hypertension (PAH), and bronchopulmonary dysplasia (BPD). Although the pathological mechanisms that cause these diseases vary, mitochondrial dynamics plays a crucial role in their occurrence and development.^318^ Notably, alveolar epithelial cells (AECs) infected with SARS‐CoV‐2 results in excessive mitochondrial division in cocultured endothelial cells.^319^, ^320^ Elucidating the molecular mechanisms and regulatory checkpoints of mitochondrial dynamics of lung disease progression will facilitate the development of potential therapies.

In recent years, the role of mitochondrial dynamics in ALI has elicited considerable interest.^8^, ^321^ *MFN2* and *OPA1* gene and protein levels are downregulated, whereas *DRP1* gene and protein levels are upregulated in the lung tissues of SD rat and mouse macrophage RAW264.7 induced by lipopolysaccharide (LPS), demonstrating mitochondrial dynamic imbalance, and largely enhancing mitochondrial fission.^8^, ^322^ The transcription of *MFN1*, *MFN2*, and *OPA1* genes of mitochondrial fusion‐related factors is significantly downregulated under LPS stress in primary murine type II AECs.^323^ Cigarette smoke extract (CSE) triggers mitochondrial fusion or fission imbalance and exacerbates mitochondrial oxidative stress and dysfunction, ultimately leading to pulmonary microvascular endothelial cell apoptosis and barrier dysfunction by promoting DRP1^S616^ phosphorylation, mitochondrial translocation, and tetramerization and downregulation of MFN2.^149^ These results indicate that changes in mitochondrial dynamics in alveolar macrophages, AECs and pulmonary microvascular endothelial cells during ALI/ARDS are characterized by reduced mitochondrial fusion and enhanced mitochondrial fission. Mitochondrial dynamic imbalances are closely related to the pathogenesis of COPD, manifested by enhanced mitochondrial fission and reduced mitochondrial fusion. CSE induces mitochondrial fragmentation by decreasing MFN2 and increasing DRP1 expression in A549 cells of alveolar epithelium^324^ In addition, biomass‐related particulate matter with a diameter of 2.5 μm or less (PM_2.5_) increases DRP1 phosphorylation, reduces MMP, and increases mitochondrial ROS in 16HBE cells, leading to mitochondrial dysfunction.^325^ Common environmental pollutants, such as PM_2.5_, cigarette smoke (CS), and allergens can trigger asthma. The expression of OPA1 and MFN1 is significantly inhibited, whereas the expression of DRP1 and FIS1 is enhanced in the presence of the highest dose PM_2.5_, resulting in fragmented and scattered mitochondria.^2^ Airway smooth muscle cells isolated from patients with moderate asthma are more sensitive to CSE than nonasthmatic samples, exhibiting decreased MFN2 expression and function, whereas DRP1‐mediated mitochondrial fission is enhanced.^160^ Interestingly, in vitro, the loss of DRP1 in lung epithelial cells reduces mitochondrial fission and enhances proinflammatory pathway in answer to house dust mite (HDM) and increased airway inflammation, hyper‐responsiveness, epithelial cell death, and differential mucin transcription in vivo.^326^ Overall, high doses of PM_2.5_, CS, and HDM enhance mitochondrial fission and reduce mitochondrial fusion, suggesting that changes in mitochondrial dynamics are mainly manifested by mitochondrial fission in asthma. Different cell types in PF exhibit varying levels of mitochondrial dynamic imbalance. Mitochondrial fusion is enhanced and fission is reduced in type II AECs by contrast, mitochondrial fission is increased, and fusion is decreased in fibroblasts during PF. Type II AECs from patients with IPF have enlarged and swollen mitochondria.^327^ These patients also show higher *MFN2* mRNA expression levels than healthy controls.^328^ DRP1 and MFF induced by stiff matrix promote mitochondrial fission in lung fibroblasts, and the DRP1/MFF pathway is activated in fibrotic lung myofibroblasts in bleomycin‐induced mouse PF and human IPF patients.^329^ Hypoxia facilitates pulmonary smooth muscle dysfunction through ER stress mediated by DRP1‐induced mitochondrial fragmentation and aggravates PAH. DRP1‐mediated mitochondrial fission increases the collagen production and proliferation rates of right ventricular fibroblasts in monocrotaline‐induced PAH.^330^ Hyperoxia significantly downregulates the expression of MFN1 and MFN2 and upregulates the expression of DRP1 in rat AECII cells RLE‐6TN compared with normoxia in BPD.^331^ Although mitochondrial dynamics has not been thoroughly studied in the occurrence and development of PAH and BPD, mitochondrial fission is mainly enhanced, whereas mitochondrial fusion is reduced in PAH and BPD.

Mitophagy (autophagy of damaged mitochondria) maintains cellular homeostasis by controlling mitochondrial dynamics and function in pulmonary sepsis.^332^ LPS enhanced PINK1/Parkin‐mediated mitophagy, increased DRP1 expression, decreased MFN2 and OPA1 expression, and mitochondrial swelling and fragmentation in endotoxin‐induced ALI, revealing that LPS exposure boost fission of damaged mitochondria segments, thereby facilitating the clearance of mitophagy.^333^ Increased mitochondrial fission and Parkin/PINK1‐dependent mitophagy promote the anticancer activity of celastrol and erastin in combination, providing new insights into the treatment of non‐small cell lung cancer.^334^ Vitamin D3 inhibits mitochondrial fission and mitophagy by downregulating the expression of DRP1, MFF and BNIP3, thereby alleviating TNF‐α‐induced lung epithelial cell inflammation.^335^ IL‐17A induces mitochondrial dysfunction in type II AECs by interfering mitochondrial dynamics, inhibits mitophagy mediated by PINK1, and leads to apoptosis of type II AECs, thus promoting PF.^336^ FIS1 enhances mitophagy by inducing mitochondrial fission, thereby promoting stemness of human lung cancer stem cells.^337^ CS exposure induces lung endothelial damage by increasing mitochondrial fission and causing abnormal mitophagy.^338^ Acute ozone exposure induced airway inflammation and bronchial hyperresponsiveness in mice by promoting mitochondrial fission and enhancing mitophagy.^339^ The above studies suggest that mitochondrial fission contributes to mitophagy in pulmonary diseases, but the details of the interaction between mitochondrial dynamics and mitophagy remain to be further explored.

In conclusion, mitochondrial dynamic imbalance is widespread in common lung diseases, such as ALI/ARDS, COPD, asthma, PF, PAH, and BPD (Table 4), suggesting that mitochondrial fusion or fission plays an important regulatory role in these diseases. At the same time, mitochondrial autophagy also plays an important role in the occurrence and development of pulmonary diseases. Although the exact mechanisms and regulatory pathways of mitochondrial fission/fusion and mitophagy remain to be explored further, they provide attractive targets for therapeutic intervention in these pulmonary diseases by inhibiting mitochondrial fission or increasing mitochondrial fusion to restore dynamic equilibrium.

**TABLE 4:** The role of mitochondrial dynamics in pulmonary disease.

Pulmonary disease	Mitochondrial dynamics	Reference(s)
ALI/ARDS	MFN1/2, OPA1↓, DRP1↑→Fusion↓, Fission↑	^333^, ^338^, ^340^
COPD	MFN2↓, DRP1↑→Fusion↓, Fission↑	^341^, ^342^
Asthma	MFN1/2, OPA1↓, DRP1, FIS1↑→Fusion↓, Fission↑	^343^, ^344^
PF	MFN2↑→Fusion↑ (AECs)	^345^
	DRP1, MFF↑→Fission↑ (fibroblasts)	^346^
PAH	DRP1↑→Fission↑	^347^, ^348^
BPD	MFN1/2↓, DRP1↑→Fusion↓, Fission↑	^349^

Abbreviations: ALI/ARDS, acute lung injury/acute respiratory distress syndrome; BPD, bronchopulmonary dysplasia; COPD, chronic obstructive pulmonary disease; PAH, pulmonary arterial hypertension; PF, pulmonary fibrosis.

## 4 CONCLUSIONS AND FUTURE PROSPECTS

Mitochondrial dynamics plays a crucial role in many key cell functions (e.g., energy production, apoptosis, migration, and biosynthesis) and the maintenance of cell vitality under stress. The entire balance of mitochondrial fusion and fission plays a crucial role in regulating mitochondrial dynamics and cell homeostasis, also requires mitophagy to coordinate and maintain the homeostasis and function. In this review, we mainly elucidate the paradigms of mitochondrial fission and fusion, especially the key regulated proteins, analyze the close relationship between mitochondrial dynamics and mitophagy, summarize their roles and potential therapeutic targets in CVDs, neurodegenerative disease, tumor, and pulmonary diseases, especially some contradictory roles. DRP1 is a key regulated protein in mitochondrial fission, so DRP1 inhibitors have some therapeutic effects in some diseases with abnormal mitochondrial division,^347^, ^348^ but treatment with a DRP1 inhibitor Mdivi‐1 in pigs did not reduce MI size or preserve cardiac function.^70^, ^129^ OPA1 and MFN1 cooperate to achieve mitochondrial membrane fusion, while has a broader role in addition to mitochondrial fusion, especially its role in mitophagy.^75^, ^350^ Although loss of mitophagy has been observed in various diseases, it seems conceivable that aberrantly overactivated mitophagy could also be detrimental and may ultimately lead to cell death and drug resistance. Mitochondrial dynamic imbalance is widespread in above mentioned diseases, suggesting that mitochondrial fusion or fission plays an important regulatory role in these diseases. The information gained from this study will add to our understanding of the relationship amongst mitochondrial fusion/fission, morphology and dysfunction in common diseases. Although the exact mechanisms and regulatory pathways of mitochondrial fission and fusion should be explored further, they provide attractive targets for therapeutic intervention. Furthermore, we describe multiple drugs and interventions that target mitochondrial dynamics or mitophagy for the treatment of these diseases. Although the molecular mechanisms of mitochondrial dynamics and their relationships with diseases remain to be elucidated. Therapies restoring balanced mitochondrial dynamics are promising tools for reducing cellular dysfunction, tissue degeneration, and function. Therefore, the development and design of pharmacological therapies targeting mitochondrial dynamics are expected to benefit in the treatment of these diseases.

Mitochondria are highly heterogeneous organelles with strict tissue and cell specificity, which result in different cell responses under different stress stimuli likely reflecting sets of complex interactions with other pathways specific to each host cell.^254^ In different tumor cells, mitochondrial fusion can inhibit tumor development and support tumor cell survival. Drug resistance is related not only to mitochondrial fission but also to mitochondrial fusion. Meanwhile, increased mitochondrial fission is closely correlated with the onset and development of cancer and is related to antitumor immunity. Ser637 is an inhibitory phosphorylation site of DRP1, but in podocytes, Drp1 phosphorylation at Ser637/656 (human/rat) promotes mitochondrial fission in response to high glucose conditions.^9^, ^35^ Different forms of the same protein play different roles in mitochondrial dynamics, such as L‐OPA1 and S‐OPA1, and their roles in various diseases need to be comprehensively explored.^8^ In addition to tissue and cell specificity, mitochondria have spatiotemporal characteristics. In ALS, mitophagy may provide neuroprotection in the beginning stages of disease progression, but if sustained for a long time, elevated mitophagy can aggravate neuron damage due to the enhanced depletion of mitochondria.^84^ BNIP3 protein levels are upregulated at the early premalignant stages of various human solid cancers, including pancreatic cancer and breast cancer, but frequently decrease when these tumors become invasive.^309^

Given the heterogeneity of mitochondria, how to properly regulate mitochondrial dynamics and mitochondrial autophagy that should be investigated. Therefore, elaborating the physiological regulation mechanism of mitochondria under various stimuli is crucial because physiologically relevant stimulation arise from mitochondrial dynamics or mitophagy can minimize the side effects of treatments and produce optimal therapeutic effects. Thus, future research should focus on the following problems: ① Whether the mitochondrial dysfunction observed is a cause or is a secondary effect of other pathogenic processes in some common diseases, including neurodegenerative disorders and some cancers should be investigated.^240^ ② How cells maintain a particular balance among fusion, fission, and mitophagy should be determined. The coordinated role of L‐OPA1 and S‐OPA1 in pathological mitochondrial dynamics deserves an in‐depth study. Fusion depends on L‐OPA1 only, whereas S‐OPA1 is associated with mitochondrial fission.^25^ The role of small and fragmented mitochondria generated through fission in tumor cell invasion and paradoxically associated with increased cell death should be determined. ③ On the basis of the complex regulatory mechanisms of mitochondria, how to specifically target pathogenic factors in mitochondrial dynamics or mitophagy to achieve the best tuning for treatment should be investigated. In cancer, the induction or inhibition of the mitochondrial fission for the destruction of tumor cells is a great challenge. The mild induction of mitophagy prevents mitochondrial dysfunction‐initiated CVD, but the excessive activation of mitophagy results in maladaptive responses and exacerbation of CVDs.^16^

## AUTHOR CONTRIBUTIONS

H. X. Y. prepared and revised the manuscript. Y. J. W., X. Y. D., H. L., and H. L. J. drafted initial manuscript. Y. J. W. prepared the figures. X. Y. D. and H. L. prepared the tables. J. F. Z., S. Y. Z., J. C. G., L. D. S., H. T. Y. and J. L. participated in the manuscript content collation. All authors contributed to the article and approved the submitted version. Y. J. W., X. Y. D., and H. L. contributed equally to this work.

## CONFLICT OF INTEREST STATEMENT

All authors declare they have no conflict of interest.

## ETHICS STATEMENT

Not applicable.

## Data Availability

Not applicable.
